# Preoperative care of Polypoid exposed mucosal template in bladder exstrophy: the role of high-barrier plastic wraps in reducing inflammation and polyp size

**DOI:** 10.1590/S1677-5538.IBJU.2017.0196

**Published:** 2018

**Authors:** Nastaran Sabetkish, Shabnam Sabetkish, Abdol-Mohammad Kajbafzadeh

**Affiliations:** 1 Pediatric Urology and Regenerative Medicine Research Center, Section of Tissue Engineering and Stem Cells Therapy, Children's Hospital Medical Center, Tehran University of Medical Sciences, Tehran, Iran (IRI)

**Keywords:** Bladder Exstrophy, Cosmetics, Infection, Polyps

## Abstract

**Objective:**

To assess the role of high-barrier plastic wrap in reducing the number and size of polyps, as well as decreasing the inflammation and allergic reactions in exstro- phy cases, and to compare the results with the application of low-barrier wrap.

**Materials and Methods:**

Eight patients with bladder exstrophy-epispadias complex (BEEC) that had used a low density polyethylene (LDPE) wrap for coverage of the exposed polypoid bladder in preoperative care management were referred. The main complaint of their parents was increase in size and number of polyps. After a period of 2 months using the same wrap and observing the increasing pattern in size of polyps, these patients were recommended to use a high-barrier wrap which is made of polyvinylidene chloride (PVdC), until closure. Patients were monitored for the number and size of polyps before and after the change of barriers. The incidence of para-exstrophy skin infection/inflammation and skin allergy were assessed. Biopsies were taken from the polyps to identify histopathological characteristics of the exposed polyps.

**Results:**

The high barrier wrap was applied for a mean ± SD duration of 12±2.1 months. Polyps' size and number decreased after 12 months. No allergic reaction was detected in patients after the usage of PVdC; three patients suffered from low-grade skin allergy when LDPE was applied. Also, pre-malignant changes were observed in none of the patients in histopathological examination after the application of PVdC.

**Conclusion:**

Polyps' size and number and skin allergy may significantly decrease with the use of a high-barrier wrap. Certain PVdC wraps with more integrity and less evaporative permeability may be more “exstrophy-friendly”.

## INTRODUCTION

Bladder exstrophy epispadias complex (BEEC) is an uncommon congenital abnormality. The prevalence of classic bladder exstrophy is approximately 1 in 50,000 live births (1). In these affected patients, the gross appearance of the bladder template, especially in the setting of polyp formation, is a major concern for the surgeon. The exact history of these polyps has not yet been documented, since many patients are born with polyps; while some seem to form later, or at least to present growth later. Concern of premalignant lesions may rise by the polypoid manifestation of the exstrophic bladder template (2). It should be also mentioned that patients can confront with more severe epithelial injury in the presence of cystitis glandularis in polyps excised during repeat or delayed primary closure (3).

At birth, the sensitive exposed bladder mucosa should be covered with a non-adherent film (Plastic Wrap) to prevent infection and sticking of the bladder to diapers or clothing (4). Low density polyethylene (LDPE) attracted the attentions as a sustainable and environmentally acceptable plastic wrap. However, the barrier quality to oxygen, aroma, microorganisms, and flavor molecules of LDPE is not as sufficient as polyvinylidene chloride (PVdC) which is also known as Saran wrap. In spite of the fact that US Saran wrap is PVdC-free, it still appears in other forms. Nevertheless, the water vapor transmission rate (WVTR) of PVdC is significantly lower than LDPE (5, 6).

Due to the importance of applying a proper wrap during the preoperative period and the role of environmental factors in polyps' formation, we decided to evaluate the clinical outcome of two different wraps (LDPE and a high--barrier wrap which is made of PVdC) in eight BEEC cases. This type of wrap with molecules bound so tightly together is supposed to act as a high barrier against oxygen, moisture, chemicals and environmental contamination. The size and the number of polyps, infection and skin allergy of the exposed polypoid bladder area, and the histopathological changes of exposed polyps were evaluated after the application of these two wraps in preoperative period.

## MATERIALS AND METHODS

Eight patients with BEEC in the setting of polyp formation were referred for delayed bladder closure from district hospitals around the country from February 2012 to April 2014. Children with failed attempts were excluded from this study. Parents of all children complained of increase in the number and size of polyps during the application of LDPE for coverage of the ex-posed bladder before being referred to our center. However, the exact size of polyps was not measured. Considering the bladder protrusion during early growth, it may not be that easy for parents to truly determine the number and size of polyps. So, after institutional review board approval and obtaining informed consent, the same wrap was used for a period of 2 months. After confirmation of increase in the number and size of polyps while applying LDPE, we recommended the use of a high-barrier wrap which is made of PVdC in the preoperative care until the time of closure. The same area of surrounded skin was covered with both wraps in order to evaluate any possible skin allergy by the application of these plastic wraps.

This non-adherent wrap was applied for the coverage of the polypoid bladder to prevent clinging of the bladder to diapers or clothing until the patient was ready to undergo closure. The plastic wraps were removed at the time of diaper change and the bladder was irrigated with sterile saline. After wiping the bladder, a clean PVdC wrap was placed. Patients were regularly evaluated for determining the number and size of polyps. Personal interviews, monthly visits for checking the bladder plate, and photographic evaluations were made to access the status of the patients regarding the number and size of polyps as well as skin allergy to the wraps. The number of polyps was precisely evaluated by a single urologist during each follow-up. In spite of the fact that the size of polyps was unchangeable in different situations of bladder plate in a same time-point, the bladder template was examined while the patient was calm, the internal abdominal pressure was not increased, and the bladder was not notably protruded in order to decrease any measurement bias.

To evaluate the histopathological characteristics of the exposed polyps, biopsies were taken from the polyps of these patients at initial visit before the application of PVdC (during the first exa-mination under anesthesia (EUA) for bladder plate measurement) and during the bladder closure (after one year of PVdC application). H&E staining was performed and the samples were analyzed by a pathologist who was totally blind to the study.

Approximation of the symphysis pubis without osteotomy was performed in all of these children according to the previously described techniques (7, 8). Moreover, sub-urothelial polyp nucleation resection and urothelial auto-augmentation cystoplasty were performed according to our recent article (9).

## RESULTS

In this study, eight children with BEEC in the setting of polyp formation (5 boys and 3 girls) with a mean ± SD age of 4.21±1.53 years (range 1 to 7) were enrolled. In all patients LDPE was applied from birth until being referred to our center. The mean ± SD duration of applying the new high barrier wrap (PVdC) was 12±2.1 months. The incidence of skin allergy decreased after the application of PVdC. In fact, 3 out of 8 patients (37.5%) experienced a low grade skin allergy when LDPE was applied in preoperative care (mild redness and swollen skin at the point of contact with the wrap); while no skin allergy was observed in none of the participants when the wrap was replaced with PVdC (Table-1).

**Table 1:** comparison of skin allergy, number and size of polyps before and after the application of a high barrier plastic wrap.

Case number	Number		Total area(mm^2^)		Skin allergy	
	Before	After	Before	After	Before	After
**No. 1**	5	5	11.32	8.54	+	-
**No. 2**	6	5	13.35	9.21	+	-
**No. 3**	4	4	10.76	8.12	-	-
**No. 4**	5	4	10.98	6.71	-	-
**No. 5**	4	4	11.06	7.52	-	-
**No. 6**	5	4	12.13	8.09	+	-
**No. 7**	3	3	9.62	7.54	-	-
**No. 8**	5	5	11.24	9.83	-	-

**Figure 1:**
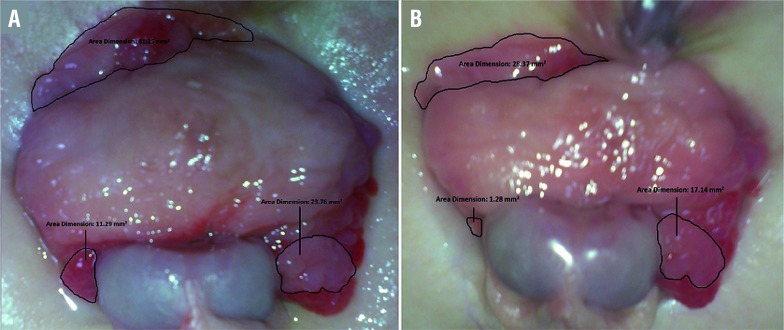
Significant decrease in the size of polyps before (A) and (B) after the application of PVdC in patient A.

**Figure 2:**
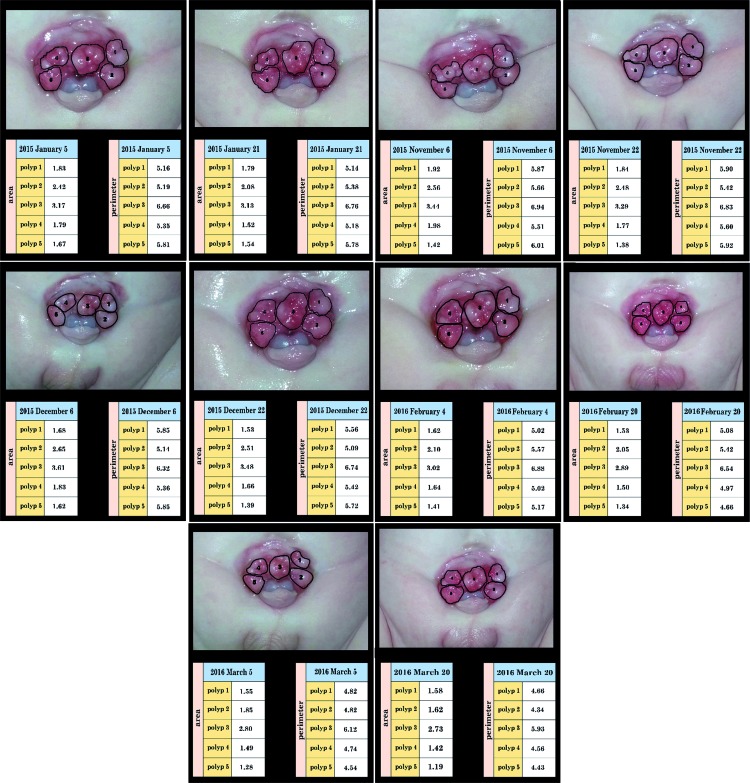
Significant decrease in the size of polyps after the application of PVdC over a period of 15 months.

**Figure 3:**
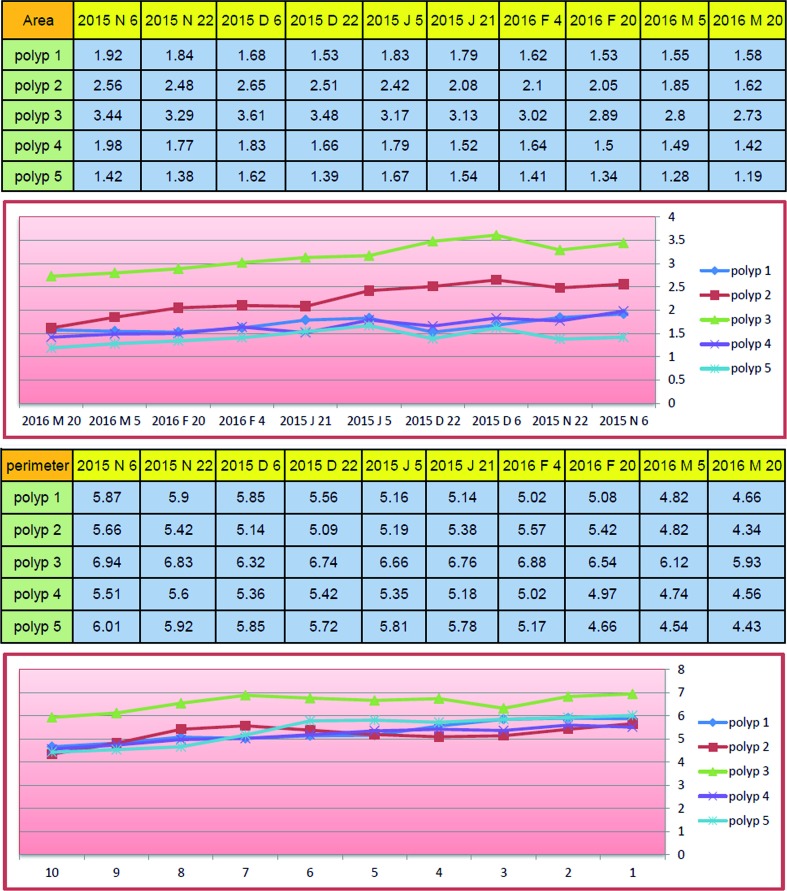
Curves of the perimeter and area of each polyp during a period of 15 months in patient B.

The number of polyps decreased in 3 patients when LDPE was replaced with PVdC (Table-1). In addition, the size of polyps decreased in all children after the application of PVdC. Figures 1–3 show the difference between the area dimensions of polyps before and after PVdC application in two different patients. Total area of polyps in each patient before and after application of PVdC is summarized in Table-1. Accordingly, even in patients with no decrease in the number of polyps, total area of polyps decreased after the application of a high barrier wrap.

Parents were more satisfied with the application of PVdC because of the absence of infection or allergic reaction as well as a decrease in the number and size of the polyps before surgical intervention.

**Figure 4:**
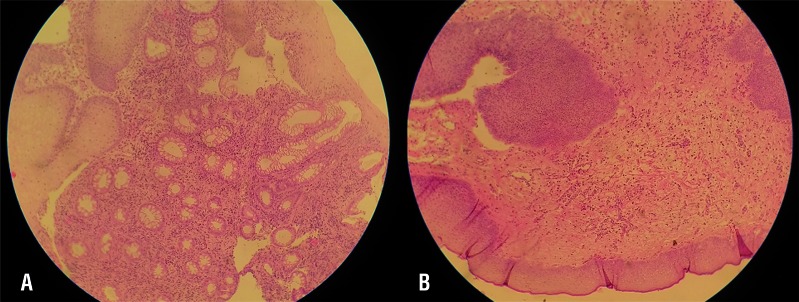
Histological evaluation of the biopsies taken from patients with LDPE (A) and PVdC (B) wrap with no malignancy or pathological changes (×40).

Histological evaluation of the biopsies taken from the polyps showed similar pathological features while applying LDPE or PVdC wrap with no significant difference. No fibrosis, edema-tous, cystitis cystic, or cystitis glandularis pattern were detected in none of the patients applying this wrap after one year before closure (Figure-4).

## DISCUSSION

The findings of the current study confirmed that the microstructural characteristics of wraps is an important factor in decreasing the size and number of polyps before surgical intervention. However, the histopathological changes are similar with the application of different wraps. Polypoid bladder template is an important concern in patients with BEC with different ages. So, the wide range of participants in this study may not confound the results.

Proponents of complete primary repair of BEEC suggested that this treatment can allow for one-stage repair as well as stimulating early bladder growth (10). Bladder polyps are one of the factors contributing to an unsuitable template for immediate closure (2). In addition, bladder exs-trophy template with multiple polyps is more susceptible to potential environmental carcinogens. So, it is clear that the bladder template should be previously covered during the preoperative period. Decrease in size and number of polyps may result in better postoperative results. In spite of the fact that excision remains superficial, large and numerous polyps may impart further weakness to the bladder after closure of the remaining mucosal defect with absorbable suture. In spite of sufficient protection during the preoperative period, polyps may form or worsen. So, application of an exstrophy-friendly coverage is obviously of great importance.

The polypoid appearance of bladder exstrophy may raise the concern of premalignant lesions. In one study, the microscopic slides of bladder exstrophy cases undergoing polyp's excision at the time of bladder wall closure were reviewed (2). In 6 out of 24 patients with primary bladder closure, two types of fibrotic and edematous polyps were observed, which were associated with overlying reactive squamous metaplasia. Comparing polyps resected during primary versus secon-dary closure, the occurrence of cystitis cystic and cystitis glandularis were higher in secondary closure. However, according to the histological evaluation of our recent study, no sign of metaplasia or dysplasia was observed in none of the patients treated with either sub-urothelial polyp nucleation resection and urothelial auto-augmentation cysto-plasty or simple excision of the polyps and bladder closure (9). It is well known that fibrous polyps are associated with profuse angiogenesis within the connective tissue stroma. However, the angiogenesis was within the normal range in the biopsies taken from both groups (9) which can allow us to apply the sub-urothelial polyp nucleation re-section and urothelial auto-augmentation cysto-plasty. The results of the present study were com-patible with the outcomes of our previous paper, in which no sign of fibrosis, edematous, cystitis cystic, or cystitis glandularis pattern was detected in the excised polyps. However, more long-term follow-ups are underway to evaluate the postoperative histopathological characteristics of these patients.

With much of the recent interest focused on improved cosmesis after the primary bladder reconstruction, little attention has been dedicated to reduce the size and number of polyps and prevent further inflammation before the operation. In several studies, it has been demonstrated that the application of a barrier dressing and frequent irrigation can preserve the bladder mucosa (11, 12). In one study in 2015, it has been demonstrated that plastic coverage during all follow-up period prevented the thickening of the mucosa and polyp formation and prevented prolonged environmental exposure of the bladder mucosa (13). In the present study, it was shown that the application of PVdC as an appropriate wrap may decrease the size and number of polyps. As an important consequence, these patients may be able to benefit from the early surgical intervention. By this technique, we can conclude that reducing the size and number of polyps before the operation may prevent further malignant potential of cystitis glandularis in polyps that are excised at secondary and delayed primary closure.

In order to reduce probable postoperative complications and achieve an acceptable continence, a careful and useful preoperative management of polyps are attainable in these selected patients and may lead to better postoperative success. Due to the fact that neonates and infants are more susceptible to preoperative complications compared with older children, we tried to evaluate the difference of PVdC and LDPE wraps in the preoperative management of polyps in patients with BEEC.

PVdC is a synthetic resin which is produced by vinylidene chloride polymerization and is a high barrier against water, aromas, oxygen, mold, bacteria, and insects. PVdC is mainly used in impermeable and flexible food wraps. PVdC is insoluble in oil and organic solvents; moreover, it is resistant to alkalizes and acids. It should be also mentioned that PVdC can release acid gas (HCl) in special conditions (exposure to gamma radiation and temperature above 125°C) (14). Copolymers of vinylidene chloride and other monomers have become popular in recent years as alternatives of PVdC with less environmental concerns. However, the amount of cling provided by LDPE plastic wraps is not as much as that PVdC plastic wraps (6, 15–17). In spite of the fact that PVdC may not be environmentally suitable due to its harmful effect after being exposed to gamma radiation or high temperatures, it may be a good choice for the coverage of the exposed bladder in patients with BEEC. On the other hand, its harmful effects do not appear in clinical conditions in which the wrap is used in room temperature and the child is not exposed to gamma radiation. These high--barrier wraps are commercially available with a low price ranging $ 2-4 (18). The results of our study revealed no harmful effects to skin by the application of PVdC while the bladders that were covered with LDPE showed mild skin allergy.

It has been confirmed that success after initial bladder closure in patients with BEEC can develop satisfactory bladder capacity and continence, reduce overall costs, and decrease the incidence of inflammation and fibrosis of the bladder (19–21). Considering the fact that the size and numbers of polyps decreased and operation could be performed earlier, more satisfactory postoperative results may be achieved in these patients. However, more evaluations in larger cohorts are needed to prove this theory. Improvement in quality of life, better postoperative managements, and decrease in the number of surgical procedures, costs, and patients discomfort are among the benefits of the current survey. Although the failure of primary closure is too complex and multifactorial, the size and number of polyps presented prior to operation can be considered as an etiology. It has been mentioned that increased inflammatory injury can occur as the result of repeat closure (6). However, by the application of a proper wrap before the operation, the number of surgeries and consequent inflammatory injury may be reduced.

To our knowledge, the current study is unique in that preoperative management of polyps in BEEC cases has not been evaluated previously. The current study has some imitations. Due to the small number of patients, statistical significance was not reached. Moreover, the postoperative outcomes of these patients were not evaluated to detect any significant difference in achieving an uneventful postoperative period. We speculated that the application of these wraps may improve the overall success of the surgical intervention and eventual continence postoperatively. However, more studies are necessary to investigate the bladder capacity, bladder growth, and histopatho-logical finding of these patients in a large group of selected patients.

## CONCLUSIONS

On the basis of the current data and our experience before and after the application of these non-adherent films, we tend to recommend a type of PVdC wrap (high-barrier) which may be preferable in preventing any further possible infection and allergic reactions of the exposed bladder region and reducing the size and number of polyps compared to other frequently used wraps.

## Abbreviations

BEEC: = bladder exstrophy epispadias complex
PVdC: = polyvinylidene chloride
LDPE: = low density polyethylene
